# Association between MMP/TIMP Levels in the Aqueous Humor and Plasma with Axial Lengths in Myopia Patients

**DOI:** 10.1155/2020/2961742

**Published:** 2020-06-10

**Authors:** Yu Yue, Yu-Wen Hsiao, Ji-Bo Zhou

**Affiliations:** ^1^Department of Ophthalmology, Shanghai Ninth People's Hospital, Shanghai Jiao Tong University, School of Medicine, Shanghai 200011, China; ^2^Shanghai Key Laboratory of Orbital Diseases and Ocular Oncology, Shanghai 200011, China

## Abstract

**Purpose:**

The present study investigated the profiles of matrix metalloproteinases (MMPs) and tissue inhibitors of metalloproteinases (TIMPs) of the aqueous humor (AH) and plasma (PL) in myopia patients, to determine whether there was an association between these levels with their axial length (AL) and to investigate if MMPs/TIMPs were regulated locally or systemically.

**Methods:**

A cross-sectional study was conducted. Thirty-nine patients (78 eyes) diagnosed with high myopia were recruited. The AL was measured using IOL Master. And the patients were divided into three groups based on their AL, Group A (AL ≤ 26 mm), Group B (26 < AL ≤ 28 mm), and Group C (AL > 28 mm). The AH in both eyes and blood samples were collected before the patients underwent implantable collamer lens surgery. In all, 78 samples of the AH and 39 samples of the PL were analyzed using MILLIPLEX map assays, followed by statistical analyses of the results.

**Results:**

There were 8 patients (16 eyes) in Group A, 22 patients (44 eyes) in Group B, and 9 patients (18 eyes) in Group C. MMP-1 (*p* = 0.014, *Β* = 0.118), MMP-2 (*p* ≤ 0.001, *Β* = 0.278), MMP-9 (*p* ≤ 0.001, *Β* = 0.019), and TIMP-1 (*p* = 0.014, *Β* = 0.062) in the AH were positively associated with the AL. MMP-1 (*p* = 0.004, *Β* = 0.001) and TIMP-1 (*p* = 0.030, *Β* = 1.171) concentrations in the PL increased linearly with longer ALs. No concentration-dependent relationship was found between MMP-2 in the PL and AL.

**Conclusions:**

There was a consistent relationship between MMP-2 in the AH and AL. AL was not consistently or substantially affected by MMP-2 in the PL, indicating myopia formation was possibly a localized process. Associations among MMP-1, MMP-9, and TIMP-1 in the AH and AL were also observed.

## 1. Introduction

The incidence of myopia has been increasing, particularly in East Asia. According to a recent cross-sectional study, the prevalence of myopia in Shanghai University students has reached 92%, with 23% of these students diagnosed with high myopia [1]. High myopia is characterized by excessive axial length (AL) elongation and can result in vision-threatening complications, including retinal detachment from peripheral tears, myopic foveoschisis, and myopic choroidal neovascularization [2].

Although several methods such as low concentrations of atropine, outdoor activities, and orthokeratology have been proposed to control myopia progression, their treatment efficacy differs by individual [3–5]. Therefore, there is an urgent need to determine the etiology of myopia, so that more efficient methods to control and prevent it can be developed.

The progression of myopia is strongly associated with scleral remodeling and excessive AL elongation. The mechanism of scleral remodeling involves the rearrangement of existing material due to microdeformations [6]. Myopic scleral changes are accompanied by excessive degradation and reduced synthesis of the scleral extracellular matrix (ECM). There is a reduction of scleral tissue during myopia development in the tree shrew [7], and a decrease in proteoglycan synthesis in primates [8]. The most important enzymes during this process are matrix metalloproteinases (MMPs), which degrade ECM proteins, and tissue inhibitors of metalloproteinases (TIMPs), which inhibit MMPs. MMPs comprise a family of 24 zinc-dependent proteases involved in physiological processes such as tissue remodeling, embryonic development, wound healing, angiogenesis, and inflammation [9]. Among MMPs, MMP-2 (72 kDa, gelatinase A) is a significant factor reported to be associated with myopia. In animal models of myopia such as tree shrews [10], chicks [11], Guinea pigs [12], and mice [13], MMP-2 is significantly upregulated. In the tree shrew, there is a selective regulation of MMP-2, TIMP-2, and TIMP-3 mRNA levels during minus-lens treatment [10]. In mice, during form-deprivation myopia, MMP-2 mRNA is elevated in a time-dependent manner [14]. Myopia development can be reduced by supplementing TIMP-2 exogenously [15].

In a previous study, we reported that MMP-2 concentrations in human AH were positively correlated with the AL [16]. Other studies have supported those results [17, 18]. However, the mechanism responsible for the upregulation of MMP-2 in myopia development remains unclear. It is assumed that the increase in MMP-2 may be associated with scleral macrophages in mice [19] and may be related to the IGF-1/STAT3 pathway in the sclera of Guinea pigs [20]. However, MMP genes are transcriptionally responsive to a wide variety of hormones, oncogenes, growth factors, and cytokines [21]. It is possible that there also might be systemic changes in the levels of MMP-2 and TIMPs, instead of ocular changes only. Among patients with inflammatory diseases such as type 1 diabetes mellitus, systemic lupus erythematosus (SLE), and juvenile chronic arthritis (JCA), higher incidences of myopia have been reported [22, 23], suggesting that systemic changes may play a role in the development of myopia.

In this study, we examined the MMP and TIMP profiles in the AH and plasma (PL) in patients with myopia but without other systemic diseases, established whether there was a concentration-dependent relationship between patients with different ALs, and investigated whether there was a relationship between MMPs and TIMPs in the PL and AH.

## 2. Materials and Methods

### 2.1. Patients and Inclusion Criteria

39 patients (78 eyes) with high myopia who underwent ICL surgery between January 2018 and October 2018 were recruited. The patient selection criteria included the following: age between 18 to 45 years, spherical equivalent (SE) of both eyes <-6.0 D, AL > 24 mm. The exclusion criteria included patients with contraindications for ICL surgery, a history of cancer, systemic inflammatory diseases (such as rheumatoid arthritis or systemic lupus erythematosus), infectious diseases (e.g., HBV, HCV, and syphilis), uncontrolled hypertension, uncontrolled diabetes, and other systemic chronic diseases. Patients with a history of glaucoma, retinal detachment, or ocular trauma were also excluded. A full inquiry about their recent oral medication, oral supplements, and eye drops being used was conducted.

Before surgery, each patient received a comprehensive ocular examination at the Department of Ophthalmology at Shanghai Ninth People's Hospital Affiliated Shanghai Jiao Tong University School of Medicine. Objective refraction was measured using an open-field autorefractor (SRW-5000; Shin-Nippon Ophthalmic Instruments, Tokyo, Japan). The AL was measured using a Zeiss IOL Master laser interferometer (Optical Biometry, IOL Master; Carl Zeiss Meditec, Jena, Germany). According to the AL, the 78 eyes were divided into three groups: Group A (AL < 26 mm), Group B (AL = 26–28 mm), and Group C (AL > 28 mm). Thirty minutes after the patient was given three drops of 1% tropicamide per eye, a dilated fundus exam was performed. The enrolled participants were classified according to the ATN grading system (based on atrophy (A), traction (T), and neovascularization (N)) [24]. One patient was classified as A0T1N0 because of an inner foveoschisis. The rest of them were classified as A0T0N0.

### 2.2. Ethics, Consent, and Permission

This study was approved by the Human Subjects Ethics Subcommittee of the Shanghai Ninth People's Hospital Affiliated Shanghai Jiao Tong University School of Medicine, and the study protocol adhered to the tenets of the Declaration of Helsinki. All participants provided written informed consent.

### 2.3. Samples

Blood samples were collected in EDTA tubes 10 min before surgery. AH samples were collected at the beginning of surgery. We aspirated the AH from the limbus by anterior chamber paracentesis using a 26-gauge needle using a 1 mL microsyringe. All samples were placed on ice immediately and then transported to the laboratory. Blood samples were transferred to a sterile 2.0 mL Eppendorf tube, then centrifuged at 1000 × *g* for 10 min at 4°C. The AH and PL were stored in 0.1 mL Eppendorf tubes at -80°C, and the samples were frozen within 4 h of collection.

### 2.4. MMP and TIMP Concentration Measurements

All samples were assayed for the total protein levels of MMP-1, MMP-2, MMP-7, MMP-9, MMP-10, TIMP-1, TIMP-2, TIMP-3, and TIMP-4 using the Luminex system (Luminex xMAP Technology; Bio-Rad, Hercules, CA, USA) with commercially available MILLIPLEX xMAP Kits (Millipore, Billerica, MA, USA). This technology uses multiplexed microsphere-based immunoassays, which apply flow cytometric resolution to spectrally measure distinct microspheres coupled with capture molecules and reporter fluorochromes bound to detection antibodies. The assays were performed following the manufacturer's guidelines and measured using a Luminex 200™ system (Bio-Rad).

The Human MMP Panel 2 Multiplex Assay (cat. no. HTIMP2MAAG-54K; Millipore) and the Human TIMP Panel 2 Multiplex Assay (cat. no. HMMP2MAG-55K; Millipore) were used. Samples were prepared by following the manufacturer's instructions. The amounts of MMPs/TIMPs (pg/mL) were calculated from the standard curves for each MMP/TIMP according to the manufacturer's instructions.

The same AH samples and PL samples were measured in each plate for an interassay control. Interassay coefficients of variation (CVs) and the range of detection are shown in supplementary materials (available here).

### 2.5. Statistical Analyses

The sample size calculation was based on the mean MMP-2 concentration in the AH and its standard deviation in myopia patients from our data base and the mean MMP-2 concentration and its standard deviation in the PL reported by Chang et al. [25]. The PASS 15.0 software (NCSS, LLC; Kaysville, UT, USA) was used with a power of 80% and a level of 95% confidence. The sample size was estimated to be 76 eyes. The SPSS 20.0 statistical software for Windows (SPSS, Chicago, IL, USA) was used for analyses. First, the Shapiro-Wilk test was conducted to check if the original data were normally distributed. Then, baseline participant-level characteristics were compared between the three groups using Fisher's exact test, Pearson's chi-squared test, the independent-samples Kruskal-Wallis test, and one-way ANOVA. The spherical equivalence (SE) of both eyes was compared using the Wilcoxon signed-rank test. Taking the correlation between both eyes into account, we performed the analyses using generalized estimating equations (GEEs) adjusted by age and sex [26], and an exchangeable correlation structure was assumed in these models. All statistical tests were two-tailed and *p* < 0.05 was considered statistically significant.

## 3. Results

AH samples were obtained from 78 eyes from 39 patients who underwent ICL surgery. In all, 16 eyes with AL < 26 mm (Group A), 44 eyes with AL = 26–28 mm (Group B), and 18 eyes with AL > 28 mm (Group C) were included. The AL (mean ± SD) was 27.15 ± 1.51 mm in the studied patients, with a mean age of 25.03 ± 6.85 years (Table 1). The mean SE of OD was −10.81 ± 2.92 diopters (D), and that of OS was −11.21 ± 4.20 D. There were 11 males and 28 females (Table 1). The three groups did not significantly differ in age (*p* = 0.976) or sex (*p* = 0.316). As expected, the ALs and SEs were statistically different (*p* ≤ 0.001) between the three groups. The SE did not significantly differ between OD and OS (*p* = 0.566). Among the 39 patients, there were 5 patients with anisometropia (differences in SE ≥ 2.0 D).

### 3.1. Analyses of MMP/TIMP Levels in the AH and PL

The concentrations of MMPs and TIMPs in AH and PL of all samples are shown in Table 2. The five-parameter logistic (5PL) model was used to estimate the concentration of each protein. As for samples unable to be analyzed using the 5PL model, we used the cubic spline model to calculate the concentrations. The concentrations of MMP-1, MMP-2, MMP-7, MMP-9, MMP-10, and TIMP-4 were much higher in the PL than in the AH, while the concentrations of TIMP-1, TIMP-2, and TIMP-3 were much higher in the AH than in the PL.

### 3.2. Differences in MMPs/TIMPs Levels in the AH and PL in the Three Groups

**Figure 1:**
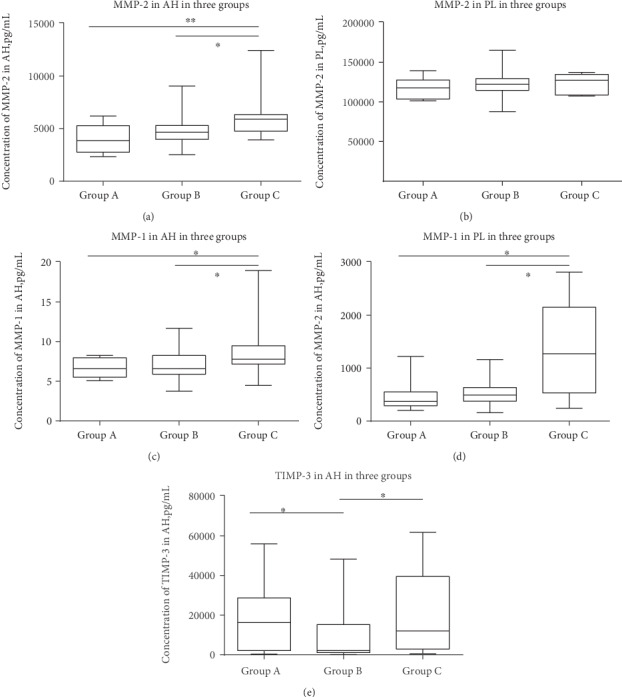
MMP-2 concentration in the aqueous humor (AH) (a) and plasma (PL) (b); MMP-1 concentration in the AH (c) and PL (d); and the TIMP-3 concentration in the AH (e). The bottom and the top of the box denote the lower and upper quantiles. The whiskers show the maximum and minimum. Differences in the protein levels among the three groups were statistically significant according to the Kruskal-Wallis test (^∗^*p* < 0.05; ^∗∗^*p* < 0.01).

The level of MMP-2 in the AH was significantly higher in Group C (*p* = 0.002) (Figure 1(a)). However, there were no significant differences in the concentration of MMP-2 in PL among the three groups (*p* = 0.192) (Figure 1(b)). Notably, the concentration of MMP-1 significantly increased in the AH (*p* = 0.002) and PL (*p* = 0.016) in Group C (Figures 1(c) and 1(d)).

Moreover, the concentration of TIMP-3 in the AH in Group B was the lowest among the three groups (Figure 1(e)). For the remaining proteins that we studied, no significant differences were found among the three groups.

### 3.3. Relationship between MMP/TIMP in the AH and AL

**Figure 2:**
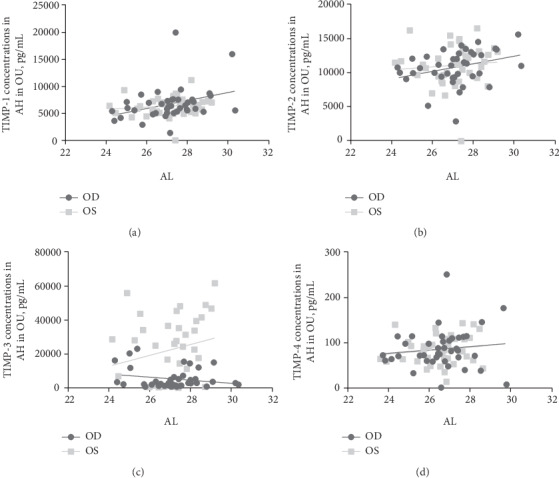
Scatter plots of concentrations of TIMP-1 (a), TIMP-2 (b), TIMP-3 (c), and TIMP-4 (d) levels in the aqueous humor. The right eye (OD) and left eye (OS) were marked differently.

Analyses of variance in the GEE showed that levels of MMP-1 (*p* = 0.014), MMP-2 (*p* ≤ 0.001), MMP-9 (*p* ≤ 0.001), and TIMP-1 (*p* = 0.014) in the AH were correlated with the AL (Table 3). In addition, the concentrations of TIMP-3 in the AH of the left eyes (OS) were higher than those in the right eyes (OD) (Figure 2).

### 3.4. Relationship between MMPs/TIMPs in the PL and AL

**Figure 3:**
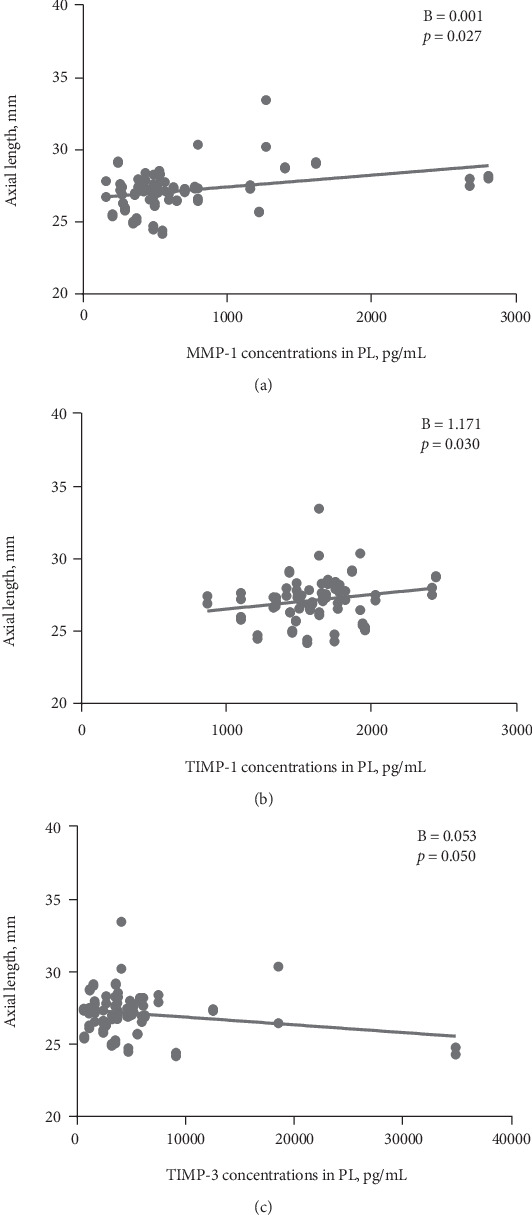
Scatter plots of concentrations of MMP-1 (a), TIMP-1 (b), and TIMP-3 (c) levels in plasma samples, plotted against the axial length.

Analyses of variance in the GEE demonstrated that concentrations of MMP-1 (*p* = 0.027) and TIMP-1 (*p* = 0.030) in the PL were associated with AL. TIMP-3 might also be associated with the AL (*p* = 0.050), but statistical significance was not found (Figure 3). However, MMP-2 in the PL was not associated with the AL (Table 3).

### 3.5. Relationship between MMPs/TIMPs in the PL and AH

Although the AH and PL were simultaneously obtained from individual patients, no significant correlation was found between the AH concentration and the PL of each protein studied.

### 3.6. Influences of Differences in Ages and Sex

According to analyses of the GEE, age (*p* = 0.995) and sex (*p* = 0.180) were not statistically associated with differences in ALs among our patients.

## 4. Discussion

There are three major findings of this study. First, the concentrations of MMP-1, MMP-2, MMP-7, MMP-9, MMP-10, and TIMP-4 were much higher in the PL than in the AH. By contrast, the levels of TIMP-1, TIMP-2, and TIMP-3 (OS) were higher in the AH than in the PL. Second, MMP-1, MMP-2, MMP-9, and TIMP-1 in the AH were positively associated with the AL. In the PL, MMP-1 and TIMP-1 concentrations increased linearly with a longer AL. However, there was no clear concentration-dependent relationship between MMP-2 in the PL and AL. Finally, no significant correlation was found between the AH concentration and the PL concentration of each protein studied.

The mechanism of myopia is not completely understood, but AL elongation and scleral remodeling are considered important processes during myopia development [7]. During this development, scleral collagen decomposition is greater than synthesis [27]. The increase in collagen degradation is regulated by MMPs and TIMPs [11, 15]. MMP-1, MMP-2, and MMP-9 are expressed in the human sclera and are potential participants in scleral remodeling [28]. Many investigators believe that MMPs and TIMPs, particularly MMP-2 and TIMP-2, are potential targets to inhibit myopia development [15, 29]. Animal studies have shown promising results; in rabbit scleral fibroblasts, MMP-2 expression is reduced and TIMP-2 expression is increased after posterior sclera reinforcement [30]; in Guinea pigs, oral intake of anthocyanin compounds reduce scleral MMP-2 expression and collagen I degradation in a form-deprivation model [29]; in Guinea pig models of lens-induced myopia, riboflavin combined with ultraviolet A irradiation results in a lower net increase in MMP-2 and a lower net decrease in TIMP-2, compared to the control group [31].

Previous studies have reported that MMP-2 is elevated in the AH and vitreous humor in myopic eyes [16, 18]; our finding that the concentration of MMP-2 in the AH was positively correlated with the AL is consistent with these results. However, we did not find a clear concentration-dependent association between MMP-2 in the PL and AL. This might imply that MMP-2 in the AH of myopia patients is not directly dependent on the MMP-2 in the PL. This possibility is consistent with a previous study that reported disparities in aqueous permeability among serum proteins without apparent size and charge preferences [32].

In this study, MMP-1 was positively correlated with AL, both in the AH and PL. MMP-9 in the AH was also positively correlated with AL. MMP-1 (also called interstitial collagenase) degrades type I, II, and III collagens. It has been considered a candidate gene in myopia formation in several studies, but the results have been conflicting [33–35]. In one study, gene polymorphism near MMP-1 was significantly associated with refractive error in an Amish family [34]. However, the same loci were not associated with myopia in an elderly cohort [33] or in the Japanese population [35], and previous studies have not quantified MMP-1 in the AH or PL from myopia patients [33–35].

As for the association between MMP-9 (also known as gelatinase B) and myopia, few previous reports were found. However, abnormal expression of MMP-9 has been observed in other ocular diseases. As a key proteolytic enzyme in the degradation of ECM [36], MMP-9 levels are upregulated in tears from patients with ocular rosacea [37] and keratoconus [38]. In the AH of diabetic macular edema patients, MMP-1 and MMP-9 concentrations are higher than normal [39]. The AH concentrations of MMP-1, MMP-2, MMP-7, and MMP-9 are also elevated in patients with retinal vein occlusion [40]. In this present study, we quantified MMP-1 and MMP-9 in AH and found a significant correlation between them with longer AL. Although the concentrations of MMP-1 and MMP-9 in the AH were lower than that of MMP-2, their possible impact on inducing scleral remodeling could not be neglected. The exact mechanism of this correlation needs further investigation.

TIMPs are the natural inhibitors of MMPs. The activity of MMPs is mainly inhibited by TIMPs via two processes. During enzyme activation, TIMP can form stable complexes with pro-MMP. In the activated MMP phase, TIMP-1 and TIMP-2 can directly form a close complex with activated MMP [36]. According to Siegwart et al., in tree shrews, TIMP-1 was downregulated during minus-lens treatment, upregulated during recovery, and then returned to normal [10]. In myopic-induced mice, Barathi et al. speculated that downregulation of TIMP-1 might play a role in preventing myopia progression [41]. In the present study, we found a positive correlation between AL and TIMP-1 levels in both the AH and the PL. The correlation between TIMP-1 in the AH and AL has been reported by Jia et al. in myopic patients [16]. The different results between experimental animal models and human subjects could be resulted from the different time when the samples are collected. The samples in the present study were collected at the stationary stage, while those of the animal models were collected during the developmental stage or the recovery stage.

TIMP-2 plays a dual role in regulating MMP-2. Exogenous administration or recombinant expression of TIMP-2 resulted in inhibition of MMP-2 activation, while endogenous physiological upregulation of TIMP-2 promoted MMP-2 activation [42]. Kudo et al. observed that TIMP-2 environment determined MT1- (membrane type 1-) MMP substrate choice between direct cleavage of MT1-MMP substrates and MMP-2 activation [43]. In tree shrews, exogenous addition of TIMP-2 significantly reduced myopia development [15]. As for TIMP-3, Siegwart et al. reported that its expression was downregulated during minus-lens treatment, then upregulated during recovery [10]. TIMP-3 binds to and inhibits MT1-MMP, and high levels of TIMP-3 strongly inhibit the activation of pro-MMP-2 [44]. These studies stated above suggest that the function of TIMP-2 could be theoretically based on a homeostatic mechanism [45]. The relative amount of MT1-MMP, TIMP-2, and TIMP-3 might be important factors in the control of pro-MMP-2 activation. In the present study, no direct correlation was found between TIMP-2 or TIMP-3 concentrations in the AH or the PL with AL. Nevertheless, further investigation concerning the relationship between TIMPs and the activity of MMP-2 may give us more information.

Moreover, we observed higher concentrations of TIMP-1, TIMP-2, and TIMP-3 (OS) in the AH than in the PL. It suggests that they could be actively transported in the AH, and/or synthesized locally. Notably, we found that the concentrations of TIMP-3 were higher in the left eyes than in the right eyes. The levels of TIMPs were measured at the same time in the same manner for each sample, and no difference was found in the levels of TIMP-1, TIMP-2, and TIMP-4. Besides, only 5 patients (12.8%) had anisometropia (SE ≥ 2.0 D). Among the participants, no significant difference was found between the SE of both eyes. One possible explanation is that we collected AH first from the OD. After completing the ICL procedure for the OD, we started collecting the AH from the OS. There was an interval of 15 min between the two eyes of the same patient. Although the underlying mechanism was not understood, it is estimated that TIMP-3 in the OS increases rapidly when the OD is being operated on. By changing the sequence of the eyes to operate, future study might be able to elucidate.

To the best of our knowledge, our study is the first to demonstrate a positive correlation between concentrations of MMP-9 in the AH and AL. And we found a positive correlation between concentrations of MMP-1 and TIMP-1 in the PL and AL. A strength of this study was that we enrolled patients at a young age, so that we could minimize the influence from other age-related systemic diseases. We also did not recruit cataract patients in the control group, AH of such eyes present with increased malondialdehyde and lipid peroxide, which may affect the levels of MMPs/TIMPs.

The limitations of our study should be discussed. First, the small number of subjects limited our results. Further studies enrolling a larger number of patients and more patients with anisometropia are needed. Second, we conducted a cross-sectional study reflecting temporal relationships between AL and MMPs/TIMPs. All patients were adults and were in the stationary stage of myopia. Therefore, we could not observe the changes of MMPs/TIMPs during myopia formation. Thirdly, our study did not have a control group with normal axial length. Anterior chamber paracentesis is an invasive procedure that raises ethical concerns if we include normal young subjects. However, age-related cataract patients with normal axial length have a large difference between our studied population in age. The possible impact of systemic diseases, including dyslipidemia and diabetes mellitus, on the plasma MMP/TIMP profile also makes cataract patients less suitable as control. Thus, we decided to compare between the groups with different axial lengths in order to minimize other influences. Correlation analyses resulted in new insights into myopia formation. The concentration of MMP-2 in the PL did not correlate with the AL. We hypothesize that in patients without systemic disease, it is possible that the changes of MMP-2 could be induced locally. When the blood-aqueous barrier is intact, the potential myopia-inducing factors including MMPs and TIMPs could be more likely to be regulated within the ocular system. Other studies have provided some evidence for this possibility. For example, optic nerve blockade does not prevent myopia induced by form deprivation [46], and restricted regions of visual field induce refractive error only in corresponding areas [47].

Nevertheless, some case reports have demonstrated that myopia can be induced by systemic diseases, such as SLE [48] and familial adenomatous polyposis [49, 50]. In both disorders, transient myopia associated with uveal effusion and a genetic defect causing abnormalities in several tissues also participate in the formation of myopia. Myopia in healthy subjects (i.e., “school myopia”) should be considered separately. We therefore hypothesize that abnormal levels of MMP-2 in the AH, which give rise to excessive ECM degradation, could more likely be induced locally, rather than systematically in healthy subjects.

## 5. Conclusions

In conclusion, we characterized MMP/TIMP profiles in the AH and PL in myopia patients. We found a positive correlation between AL and levels of MMP-1 and TIMP-1, both in the AH and PL, despite a rather low concentration of MMP-1 in the AH. A positive correlation between AL and levels of MMP-9 in the AH was also observed. Our study showed a consistent relationship between MMP-2 in the AH and AL. However, the AL was not consistently or substantially affected by MMP-2 in the PL, leading to the hypothesis that abnormal expression of MMP-2 in the AH, which is closely related to scleral remodeling, could more likely be induced locally, rather than systematically. Additional studies about the role of MMP-1 and MMP-9, the activity of MMP-2, and the homeostasis of pro-MMP-2 and TIMPs are needed, to validate these preliminary results.

## Figures and Tables

**Table 1:** Demographic characteristics of patients with a high degree of myopia, divided into three groups, based on the axial length (AL). Group A: AL < 26 mm; Group B: 26 mm < AL ≤ 28 mm; Group C: AL > 28 mm.

Clinical characteristics	A	B	C	Total	*p* value
Sex					
Female	7	15	6	28	0.316^†^
Male	1	7	3	11	
Eye					
OD	8	22	9	39	1.000^‡^
OS	8	22	9	39	
Age (mean ± SD)	24.75 ± 5.63	25.32 ± 7.71	24.56 ± 5.78	25.03 ± 6.85	0.976^§^
Axial length (mean ± SD) (mm)					
OD	25.05 ± 0.56	27.16 ± 0.50	28.92 ± 0.89	27.13 ± 1.42	≤0.001^*ξ*^
OS	25.10 ± 0.63	27.10 ± 0.50	29.13 ± 1.67	27.16 ± 1.62	≤0.001^§^
Spherical equivalent (mean ± SD), diopters					
OD	−8.21 ± 2.06	−10.75 ± 1.83	−13.26 ± 3.81	−10.81 ± 2.92	0.004^§^
OS	−8.19 ± 2.55	−11.15 ± 3.52	−14.01 ± 5.25	−11.21 ± 4.20	0.003^§^

OD: right eye; OS: left eye. ^†^Fisher's exact test. ^‡^Pearson's chi-squared test. ^§^Independent-sample Kruskal-Wallis test. ^*ξ*^One-way ANOVA test.

**Table 2:** Protein concentrations in the AH and PL.

Proteins	MW, kDa	AH concentration: *P*_50_ (*P*_25_, *P*_75_), pg/mL		PL concentration: *P*_50_ (*P*_25_, *P*_75_), pg/mL	Ratio	
		OD	OS		OD/PL	OS/PL
MMP-1	45.1	6.57 (5.66, 8.17)	7.33 (5.96, 8.41)	507.29 (367.27, 780.99)	1/100	1/100
MMP-2	72	4912.00 (4106.50, 5772.50)	4633.00 (3738.00, 5459.00)	121122.00 (113326.25, 130046.50)	4/100	4/100
MMP-7	28	369.64 (283.35, 495.15)	369.64 (253.61, 477.89)	17895.50 (10156.25, 27812.25)	2/100	2/100
MMP-9	82	19.55 (14.79, 34.43)	16.17 (13.42, 25.31)	34705.50 (27343.75, 55667.50)	0	0
MMP-10	57	68.50 (50.90, 91.35)	65.80 (48.60, 92.00)	244.21 (201.37, 319.98)	28/100	27/100
TIMP-1	28	6121.50 (5321.75, 7542.00)	6509.50 (5107.50, 7410.50)	1588.00 (1446.50, 1772.00)	385/100	410/100
TIMP-2	21.7	11155.50 (9548.25, 12878.00)	11451.00 (9515.00, 13219.75)	3114.00 (2911.50, 3384.00)	358/100	368/100
TIMP-3	30	2818.00 (1369.25, 6134.25)	24675.00 (855.19, 37349.75)	3633.50 (1822.25, 5469.50)	78/100	679/100
TIMP-4	22	78.85 (61.77, 111.30)	83.72 (60.90, 108.58)	355.37 (277.13, 425.40)	22/100	24/100

MW: molecular weight; AH: aqueous humor; PL: plasma; OD: right eye; OS: left eye; Ratio OD/PL: the ratio of median concentration in the AH of OD to that of PL; the same for Ratio OS/PL.

**Table 3:** Association between MMPs/TIMPs in the AH and PL with AL.

		GEE	
		*B*	*p* value
Concentrations in the AH	MMP-1, pg/mL	0.118	0.014
	MMP-2, ng/mL	0.278	≤0.001
	MMP-7, ng/mL	4.699 × 10^−5^	0.923
	MMP-9, pg/mL	0.019	≤0.001
	MMP-10, pg/mL	0.003	0.583
	TIMP-1, pg/mL	0.062	0.014
	TIMP-2, ng/mL	0.038	0.214
	TIMP-3, ng/mL	0.002	0.445
	TIMP-4, pg/mL	0.001	0.827

Concentrations in the PL	MMP-1, pg/mL	0.001	0.027
	MMP-2, ng/mL	0.023	0.103
	MMP-7, ng/mL	0.022	0.380
	MMP-9, ng/mL	-0.005	0.530
	MMP-10, pg/mL	0.001	0.209
	TIMP-1, ng/mL	1.171	0.030
	TIMP-2, ng/mL	0.398	0.282
	TIMP-3, ng/mL	-0.053	0.050
	TIMP-4, pg/mL	0.001	0.774

AH: aqueous humor; AL: axial length: PL: plasma; GEE: generalized estimating equation.

## Data Availability

The data have not been placed in any online data storage. The datasets generated and analyzed during the study are available upon request from the first author.

## Additional Points

The English in this document has been checked by at least two professional editors, both native speakers of English. For a certificate, please see http://www.textcheck.com/certificate/h9hXbp.

## Supplementary Materials

Supplementary Materials The interassay precision is generated from the mean of the % coefficients of variation (CVs) across two different concentrations of analytes across different assays. According to the protocol of MILLIPLEX®, the % CV should be less than 30%. The minimum detectable concentrations (MinDC) measure the true limits of detection. The % CV and MinDC of each plate are shown in supplementary materials.
