# Supplementary material for: Association between MMP/TIMP Levels in the Aqueous Humor and Plasma with Axial Lengths in Myopia Patients
Source: Biomed Res Int. 2020 Jun 10;2020:2961742. doi: 10.1155/2020/2961742 (PMC7305534; doi:10.1155/2020/2961742)
Supplement: Supplementary Materials — The interassay precision is generated from the mean of the % coefficients of variation (CVs) across two different concentrations of analytes across different assays. According to the protocol of MILLIPLEX®, the % CV should be less than 30%. The minimum detectable concentrations (MinDC) measure the true limits of detection. The % CV and MinDC of each plate are shown in supplementary materials. [file 2961742.f1.docx]

**Supplementary Materials**

The coefficient of variation and the range of detection.

Plate 1 PL

| Analyte | Chi | R^2^ | CV | MinDC (pg/mL) |
| --- | --- | --- | --- | --- |
| MMP-1 | 0.040% | 0.999 | 0.91% | 2.6 |
| MMP-2 | 0.012% | 1 | 0.40% | 5.4 |
| MMP-7 | 0.071% | 0.999 | 1.72% | 3.7 |
| MMP-9 | 0.023% | 1 | 0.62% | 1.9 |
| MMP-10 | 0.025% | 1 | 0.66% | 2.1 |

PL, plasma; CV, coefficient of variation; MinDC, minimal detectable concentration.

Plate 2. AH

| Analyte | Chi | R^2^ | CV | MinDC |
| --- | --- | --- | --- | --- |
| MMP-1 | 0.028% | 1 | 0.70% | 2.6 |
| MMP-2 | 0.059% | 0.999 | 2.51% | 5.4 |
| MMP-7 | 0.0097% | 1 | 0.26% | 3.7 |
| MMP-9 | 0.012% | 1 | 0.37% | 1.9 |
| MMP-10 | 0.020% | 1 | 0.59% | 2.1 |

AH, aqueous humor; MinDC, minimal detectable concentration.

Plate 3. AH

| Analyte | Chi | R^2^ | CV | MinDC |
| --- | --- | --- | --- | --- |
| TIMP1 | 0.032% | 1 | 1.09% | 4 |
| TIMP2 | 0.065% | 0.999 | 2.27% | 9 |
| TIMP3 | 0.033% | 1 | 1.85% | 52 |
| TIMP4 | 0.024% | 1 | 0.87% | 4 |

AH, aqueous humor; MinDC, minimal detectable concentration.

Plate 4 PL

| Analyte | Chi | R^2^ | CV | MinDC |
| --- | --- | --- | --- | --- |
| TIMP1 | 0.0060% | 1 | 0.20% | 4 |
| TIMP2 | 0.0063% | 1 | 0.22% | 9 |
| TIMP3 | 0.019% | 1 | 0.91% | 52 |
| TIMP4 | 0.0020% | 1 | 0.070% | 4 |

PL, plasma; MinDC, minimal detectable concentration.
